# Exercise interventions for patients with heart failure: an evidence map

**DOI:** 10.3389/fspor.2025.1557887

**Published:** 2025-03-28

**Authors:** Tingting Wang, Ling Ji, Jian Li, Min Zhang, Sixuan Han, Yanyan Hong

**Affiliations:** ^1^ Proctology Department, Nanjing Hospital of Chinese Medicine, Affiliated to Nanjing University of Chinese Medicine, Nanjing, China; ^2^ Nursing Department, Nanjing Hospital of Chinese Medicine, Affiliated to Nanjing University of Chinese Medicine, Nanjing, China

**Keywords:** heart failure, evidence map, systematic review, meta-analysis, exercise intervention

## Abstract

**Background and aims:**

Although exercise performance is an essential tool for managing heart failure, there is still no consensus on whether exercise prescriptions can be universally applied to all types of heart failure patients. This study aimed to describe and evaluate the evidence on exercise interventions for patients with heart failure by creating an evidence map.

**Methods:**

We searched PubMed, Web of Science, EMBASE, the Cochrane Library, China National Knowledge Infrastructure (CNKI), Wanfang Database, VIP Database to identify systematic reviews and meta-analysis. We used A Measurement Tool to Assess Systematic Reviews (AMSTAR-2) to evaluate the quality of included systematic reviews and meta-analysis. Bubble charts were employed to visualize key characteristics like intervention effectiveness, literature quality, literature quantity, and heart failure types. Matrix bubble charts were used to show the distribution of outcome indicators, exercise methods, literature quantity, and heart failure types.

**Results:**

A total of 113 systematic reviews/meta-analyses were included and over 80% of studies conclude that exercise is beneficial for heart failure patients. Three heart failure types involved varied due to different classification criteria used in the included literature. Eleven types of exercise interventions have been applied in patients with heart failure and mixed modality exercise is the exercise type with the highest attention. Existing researches focus more on the improvement of exercise capacity and cardiorespiratory function in heart failure patients. Most researchers tend to focus on conducting exercise intervention studies in HFrEF.

**Conclusions:**

The evidence map provides a visual overview of the research volume and findings on exercise interventions for heart failure patients. Exercise interventions have significant advantages for heart failure patients, but there is room for improvement in study quality, heart failure classification, and outcome indicators. Future research should focus on designing more high-quality studies to provide more high-level evidence for this field.

## 1 Introduction

Heart failure (HF) is the final stage of various cardiovascular diseases (1). It is a clinical syndrome defined by common symptoms, such as dyspnea, ankle swelling, and fatigue, which may be accompanied by signs like elevated jugular venous pressure, pulmonary crackles, and peripheral edema. These manifestations are typically the result of structural and/or functional heart abnormalities, leading to reduced cardiac output and/or increased intracardiac pressures, either at rest or under stress (2). Heart failure is diagnosed only when these symptoms are evident. Research (3) shows that approximately 37.7 million people globally suffer from heart failure and the prevalence and mortality rate of heart failure continue to rise. Symptoms such as dyspnea, fatigue, and fluid retention progressively worsen, continuously challenging the heart function of patients while also diminishing their exercise capacity. Patients often experience anxiety, depression, and a decline in quality of life (2).

Exercise performance is an essential tool for managing heart failure. Studies have shown (4, 5) that exercise rehabilitation can improve cardiac function, enhance exercise tolerance, and improve quality of life. Exercise rehabilitation has been recommended with Class IA evidence in heart failure management guidelines internationally. Since the concept of cardiac rehabilitation (6) was introduced, exercise-based rehabilitation and its effects have become a research hotspot and several studies have been published. Although the benefits of exercise are well documented for people with chronic and stable heart failure, there is still no consensus on whether exercise prescriptions can be universally applied to all types of heart failure patients (7, 8). What types of exercise have been researched? Which outcomes in heart failure patients have been improved? Is it possible to summarize the optimal exercise prescriptions for different types of heart failure based on published evidence? This study aims to explore this issue through the use of an evidence map.

Evidence Maps (9–13) are new synthesis tools which involve searching systematically and characterizing existing research on a topic of interest for the identification of knowledge gaps and future research needs. Compared to traditional systematic reviews, evidence mapping offers a broader perspective and more diversified research types (14).The bubble chart is the highlight of the evidence map. It visually presents multidimensional information on the quantity of evidence, research quality, and intervention effects through bubble size, color, and position. This method is superior to traditional tables or bar charts, allowing readers to quickly identify key patterns and trends (15).

Given the limitations of existing clinical trials (16–18) in terms of sample size, methodological heterogeneity, and consistency of conclusions, this study aims to provide a comprehensive conclusion with a higher level of evidence by integrating systematic reviews/meta-analyses, filling the gap in the current literature regarding the overall assessment of exercise intervention effects in heart failure patients. Systematic reviews/meta-analyses have always been considered the gold standard for evaluating the effectiveness of interventions and they are at the top of the research quality pyramid (19).

From a clinical perspective, systematically screening and organizing evidence on exercise interventions for heart failure is essential for practitioners to carry out in-depth clinical research. From a research perspective, there is a contradiction between the increasing number of studies on exercise interventions for heart failure and the lack of high-quality evidence. Many studies suffer from a lack of innovation and merely replicate existing research, which makes it difficult for existing evidence to meet the ever-evolving demands of clinical practice. Therefore, it is necessary to comprehensively evaluate the existing evidence at a macro level, identify gaps in research, and reduce the blindness of future studies to prevent the waste of limited resources.

This study aims to provide more comprehensive, high-quality evidence for clinical researchers and policymakers by summarizing and analyzing systematic reviews/meta-analyses on exercise interventions for heart failure, along with a concomitant narrative that serves as a reference for future primary research and systematic reviews/meta-analyses. The uniqueness of this study lies in visualizing the population, effects, and evidence quality of exercise interventions for heart failure patients through an evidence map, providing an intuitive basis for clinical decision-making.

## 2 Methods

### 2.1 Electronic search

The following electronic databases were systematically searched for in literature published from their dates of inception to 30 August 2024: PubMed, Web of Science, EMBASE, and Cochrane Library, China National Knowledge Infrastructure (CNKI), Wanfang Database, VIP Datebase. In addition, a snowball method was used to track relevant systematic reviews/meta-analyses and the references of the included studies.

The search terms used were “heart failure”, “exercise” and “systematic review” or “meta-analysis”without language restrictions (Supplementary 1).

### 2.2 Inclusion and exclusion criteria

Search results were independently reviewed for eligibility by two independent researchers (Tingting Wang and Ling Ji), with discrepancies resolved by a third researcher (Min Zhang). Non-native language literature was fully translated by professional translators and reviewed for accuracy by two independent researchers (Tingting Wang and Jian Li) to ensure the correctness of data extraction. Studies were included based on the following criteria.

Inclusion Criteria:

(i) Population: patients with confirmed heart failure.
(ii) Interventions: all trials were included regardless of the type of exercise and control intervention. Trials that used exercise as an adjunctive treatment were also included.
(iii) Study type: systematic review or meta-analysis.

Exclusion Criteria:

(i) Duplicate publications.
(ii) Insufficient data, or articles where data could not be extracted.
(iii) Full text not available through any means.
(iv) Animal studies.

### 2.3 Quality assessment

The AMSTAR-2 scale (A Measurement Tool to Assess Systematic Review-2) was used to evaluate the methodological quality of the included systematic reviews/meta-analyses (20, 21). Which consists of 16 items(critical items: 2, 4, 7, 9, 11, 13, 15), each rated as “Yes”, “Partial Yes”, or “No”. The quality assessment results were categorized into four levels: “high” means no or one nonkey item missing,“moderate” means more than one nonkey item missing, “low” means one key item with or without nonkey items missing, and “critically low” means more than one key item with or without nonkey items missing (22).

A stacked bar chart was used to demonstrate the methodological quality of included studies. Two reviewers (Tingting Wang and Ling Ji) independently evaluated each study and rated the studies according to the AMSTAR-2 tool. Discrepancies in risk assessment were resolved by consensus and, if required, consultation with a third reviewer (Min Zhang).

### 2.4 Data extraction

Duplicate references were removed using NoteExpress software. Based on predefined inclusion and exclusion criteria, titles and abstracts were reviewed to exclude irrelevant studies. The remaining articles were downloaded and read in full for further screening, with the final selection of studies determined for inclusion. Two reviewers (Tingting Wang and Ling Ji) independently extracted data using a predesigned table included: publication year, first author's country, type of heart failure, exercise methods, outcome indicators, and risk of bias assessment tools.

### 2.5 Data synthesis and analysis

Microsoft Excel 2020 was used to extract the characteristics of the included systematic reviews/meta-analyses. We designed a bubble plot to display multidimensional information. (a) The *x*-axis represented the classification of the author's conclusions (“Beneficial”, “No differential effect,”). (b) The *y*-axis represented the evaluation results of AMSTAR-2. (c) The size of the bubbles and the numbers on them indicated the number of primary studies contained in the systematic reviews/meta-analyses. (d) The colors of the bubbles indicated different classification of heart failure.

Matrix Bubble Chart was drawn to present Intervention-Outcome indicator evidence distribution. (a) The *X*-axis in the bubble matrix chart represents different outcome indicators. (b) The *Y*-axis displays different types of exercise interventions. (c) The size of the bubbles reflects the number of studies. (d) The color indicates different heart failure types.

## 3 Results

### 3.1 Selected studies

**Figure 1:**
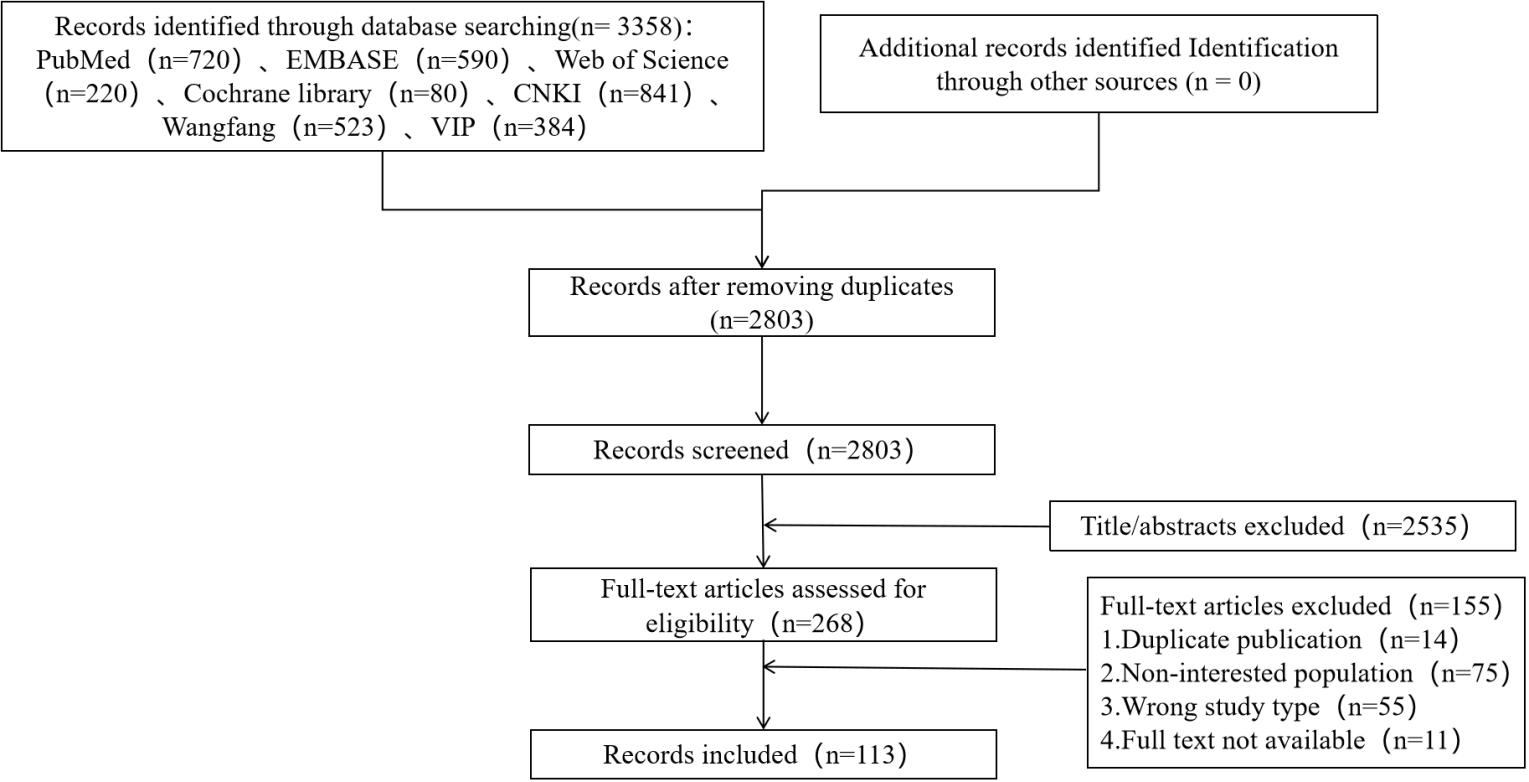
PRISMA flow diagram of the studies selection.

A total of 113 studies were included in this evidence mapping. A flow diagram of study selection is presented in Figure 1.

### 3.2 Basic characteristics of included studies

A total of 113 articles were included, comprising 13 Chinese articles (11.50%) and 100 English articles (88.50%). The included literature came from 15 different countries. The top three countries in terms of the number of publications were China (45 articles, 39.82%), Australia (16 articles, 14.16%), and the United Kingdom (13 articles, 11.50%).

The studies included different types of heart failure due to varying classification standards (23). These included:

(a) Acute heart failure (AHF): 2 articles (1.77%),
(b) Chronic heart failure (CHF): 12 articles (10.62%),
(c) Heart failure with preserved ejection fraction (HFpEF): 12 articles (10.62%),
(d) Heart failure with mid-range ejection fraction (HFmrEF): 1 article (0.88%),
(e) Heart failure with reduced ejection fraction (HFrEF): 30 articles (26.55%),
(f) HFmrEF + HFpEF: 2 articles (1.77%),
(g) HFpEF + HFrEF: 8 articles (7.08%),
(h) HFrEF + HFmrEF: 11 articles (9.73%),
(i) NYHA class I-III: 11 articles (9.73%),
(j) NYHA class II-III: 4 articles (3.54%),
(k) Unclassified heart failure: 20 articles (17.70%).

Exercise interventions included 11 main types, with mixed exercise (combining two or more types of exercise) being the most common (42.48%, 48 articles) (24). Other types included:

(a) Aerobic exercise (25): 6 articles (5.31%),
(b) Interval training (26): 19 articles (16.81%),
(c) Resistance training (27): 5 articles (4.42%),
(d) Endurance training (28): 1 article (0.88%),
(e) Traditional exercises (29): 10 articles (8.85%),
(f) Aquatic exercise (30): 4 articles (3.54%),
(g) Exercise games (31): 1 article (0.88%),
(h) Inspiratory muscle training (32): 2 articles (1.77%),
(i) Rehabilitation training without specified exercise type (33): 16 articles (14.16%),
(j) Digital remote rehabilitation exercise (34): 1 article (0.88%).

The main outcome indicators included:

(a) Cardiopulmonary function: 61 articles (53.98%),
(b) Exercise capacity: 89 articles (78.76%),
(c) Quality of life: 48 articles (42.48%),
(d) Inflammatory markers: 5 articles (4.42%),
(e) Traditional Chinese Medicine symptom scores: 1 article (0.88%),
(f) Psychological status: 4 articles (3.54%),
(g) Other indicators (mortality, hospitalization rate, morbidity): 12 articles (10.62%).

Compared to the control group, 95 studies concluded that the intervention group was superior, while 18 studies found no significant difference between the two groups. Of the 113 studies, 104 provided detailed descriptions of the intervention time, intensity, and frequency, and 30 articles mentioned the setting for exercise interventions, which included medical institutions, hospitals, outpatient clinics, homes, rehabilitation centers, gyms, and outdoor environments.

The researcher who has published the most articles on this topic is Mansueto Gomes Neto (35) from Brazil, who investigated six different types of exercise interventions for heart failure patients. The basic characteristics of the included studies are summarized in Table 1.

**Table 1:** Basic characteristics of included studies.

Basic characteristics	Number of articles	Percentage
Publication year		
Before 2010	9	7.96%
2011–2020	59	52.21%
2021–2024	45	39.82%
First author's country		
China	45	39.82%
Australia	16	14.16%
United Kingdom	13	11.50%
Brazil	12	10.62%
United States	8	7.08%
Other countries (10 countries)	19	16.81%
The type of heart failure		
Acute heart failure (AHF)	2	1.77%
Chronic heart failure (CHF)	12	10.62%
Heart failure with preserved ejection fraction (HFpEF)	12	10.62%
Heart failure with mid-range ejection fraction (HFmrEF)	1	0.88%
Heart failure with reduced ejection fraction (HFrEF)	30	26.55%
HFmrEF + HFpEF	2	1.77%
HFpEF + HFrEF	8	7.08%
HFrEF + HFmrEF	11	9.73%
NYHA Class I-III	11	9.73%
NYHA Class II-III	4	3.54%
Unclassified heart failure	20	17.70%
The type of intervention		
Aerobic exercise	6	5.31%
Interval training	19	16.81%
Resistance training	5	4.42%
Endurance training	1	0.88%
Traditional exercises	10	8.85%
Aquatic exercise	4	3.54%
Exercise games	1	0.88%
Inspiratory muscle training	2	1.77%
Mixed modality exercise	48	42.48%
Digital remote rehabilitation	1	0.88%
Rehabilitation without specified exercise type	16	14.16%
Outcome indicators		
Cardiopulmonary function	61	53.98%
Exercise capacity	89	78.76%
Quality of life	48	42.48%
Inflammatory markers	5	4.42%
TCM symptom scores	1	0.88%
Psychological status	4	3.54%
Other (mortality, hospitalization rate, morbidity)	12	10.62%
Bias risk assessment tools		
Cochrane bias risk tool	60	53.10%
Jadad scale	10	8.85%
JBI score	3	2.65%
PEDro scale	25	22.12%
TESTEX scale	13	11.50%
Downs and black quality index	1	0.88%
Delphi list score	1	0.88%
Intervention details		
Described intervention time, intensity, frequency	104	92.04%
Not described	9	7.96%
Efectiveness evaluation		
Intervention group superior	95	84.07%
No significant difference	18	15.93%

### 3.3 Methodological quality of included studies

**Figure 2:**
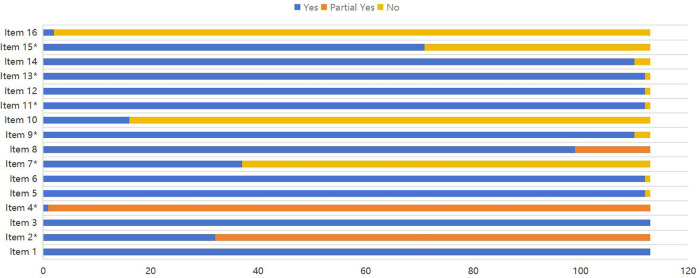
Methodological quality of the included systematic reviews. Items: 1. Did the research questions and inclusion criteria for the review include the components of PICO?. 2*. Did the report of the review contain an explicit statement that the review methods were established prior to the conduct of their review, and did the report justify any significant deviations from the protocol?. 3. Did the review authors explain their selection of the study designs for inclusion in the review? 4*. Did the review authors use a comprehensive literature search strategy?. 5. Did the review authors perform study selection in duplicate?. 6. Did the review authors perform data extraction in duplicate?. 7*. Did the review authors provide a list of excluded studies and justify the exclusions?. 8.Did the review authors describe the included studies in adequate detail?. 9*. Did the review authors use a satisfactory technique for assessing the risk of bias (RoB) in individual studies that were included in the review?. 10. Did the review authors report on the sources of funding for the studies included in the review? 11*. If meta-analysis was performed did the review authors use appropriate methods for statistical combination of results?. 12.If meta-analysis was performed, did the review authors assess the potential impact of RoB in individual studies on the results of the meta-analysis or other evidence synthesis?. 13*. Did the review authors account for RoB in individual studies when interpreting/discussing the results of the review?. 14. Did the review authors provide a satisfactory explanation for, and discussion of, any heterogeneity observed in the results of the review?. 15*. If they performed quantitative synthesis did the review authors carry out an adequate investigation of publication bias (small study bias) and discuss its likely impact on the results of the review?. 16. Did the review authors report any potential sources of conflict of interest, including any funding they received for conducting the review?. *Asterisk represents key entries.

The quality assessment results showed that 6 articles (5.31%) were high quality, 9 articles (7.96%) were moderate quality, 77 articles (68.14%) were low quality, and 21 articles (18.58%) were very low quality. All studies followed the “PICO” principle and explained the reasons for including specific study designs in the systematic reviews. Thirty-two studies had been registered or published in advance with research plans. Only one study supplemented the database search with clinical trials or research registration platforms, gray literature, and other sources. Only two studies described the funding sources, and none declared any conflicts of interest. These results are shown with stacked bar chart in Figure 2.

### 3.4 Evidence presentation

Bubble charts were used to reflect the relationships between multiple variables. This study utilized three bubble charts to present the basic characteristics of the included studies. The first bubble chart visualizes the overall effectiveness of exercise interventions for heart failure patients. The second chart shows the differences in key features between different exercise types and the most frequently measured outcome indicators. The third chart shows the differences in key features between different heart failure types and different exercise modalities.

#### 3.4.1 Intervention effectiveness evidence distribution

**Figure 3:**
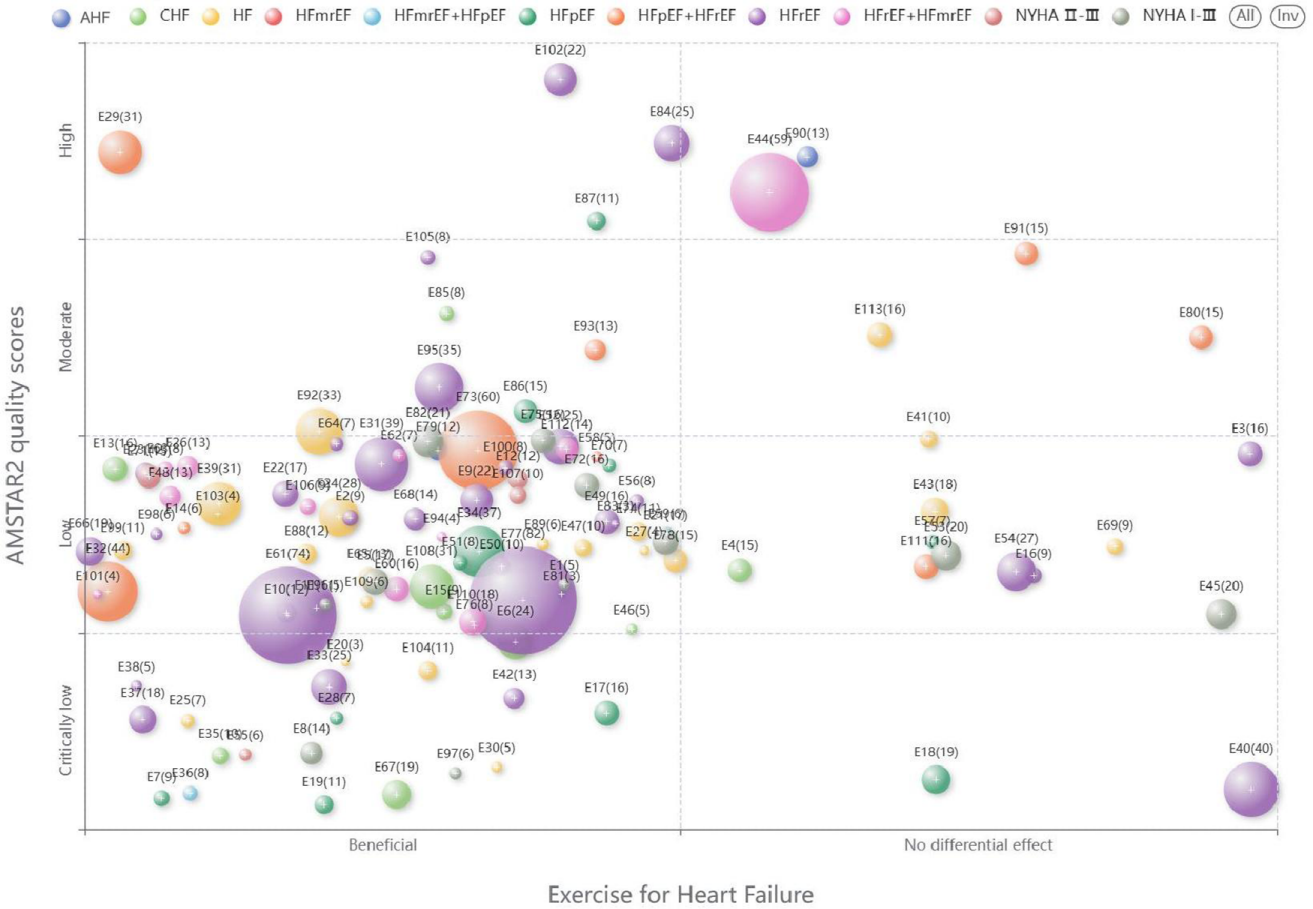
Evidence Map of exercise interventions for heart failure.

Among the 113 included studies, 95 concluded that the intervention group was superior to the control group, while 18 concluded that there was no significant difference between the groups. The *X*-axis in the bubble chart represents the final conclusion of each study, divided into “Beneficial” and “No differential effect,” while the *Y*-axis represents the methodological quality of the studies, categorized as “High,” “Moderate,” “Low,” or “Very Low.” The bubbles are labeled with “E + number” to represent different studies, and their size corresponds to the number of studies included in the systematic review/meta-analysis. The color of the bubbles represents different types of heart failure. The distribution is shown in Figure 3.

#### 3.4.2 Intervention-outcome indicator evidence distribution

**Figure 4:**
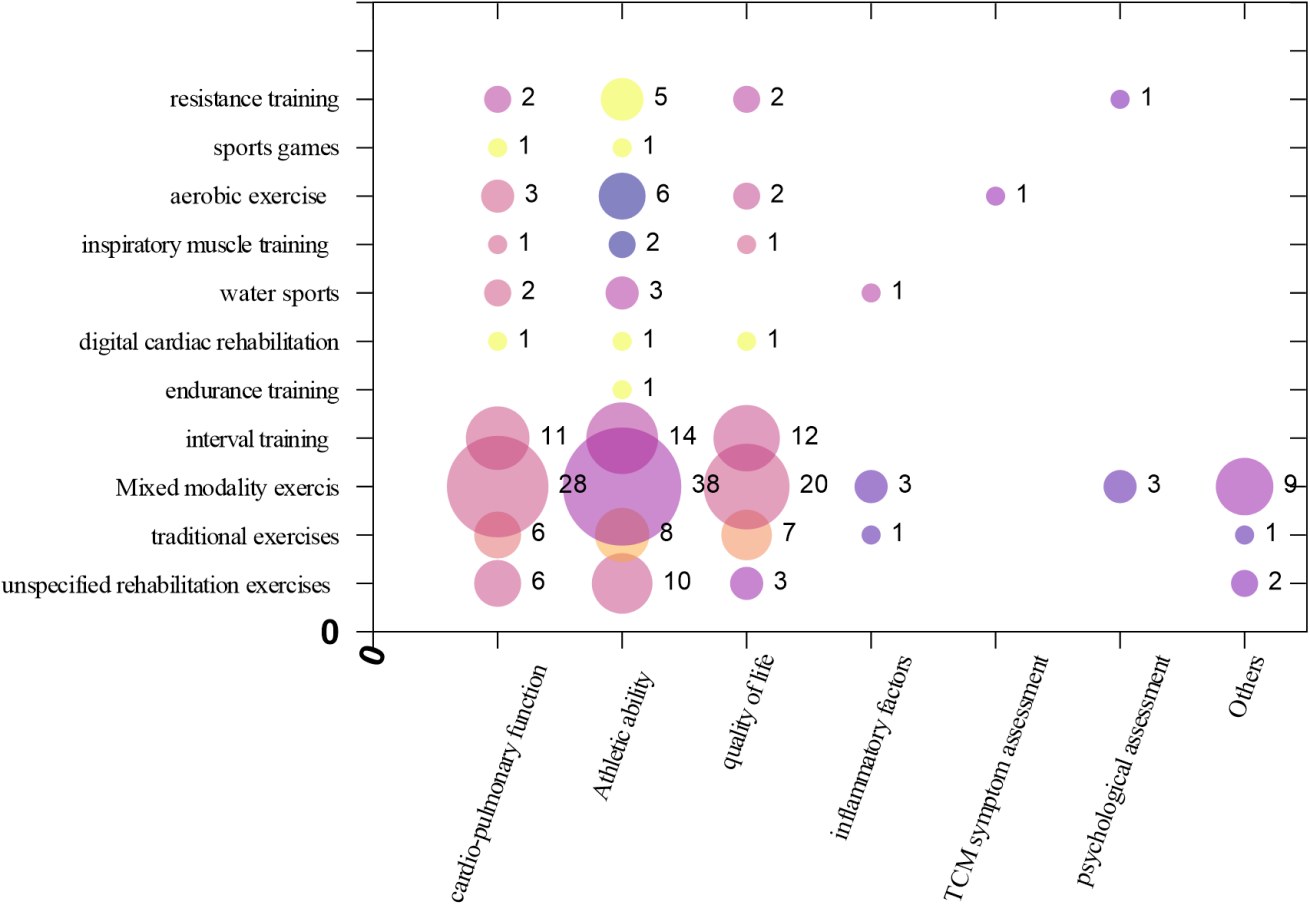
Evidence Map of outcome indicators for exercise interventions in heart failure.

The outcome indicators most commonly measured in the studies were exercise capacity (89 articles), cardiopulmonary function (61 articles), and quality of life (48 articles). Fewer studies focused on other outcome indicators such as inflammatory markers (5 articles), psychological status (4 articles), TCM symptom scores (1 article), and other outcomes such as mortality, hospitalization rate, and morbidity (12 articles). The *X*-axis in the bubble chart represents different outcome indicators, while the *Y*-axis represents different types of exercise interventions. The size of the bubbles reflects the number of studies. This distribution is shown in Figure 4.

#### 3.4.3 Intervention-population classification evidence distribution

**Figure 5:**
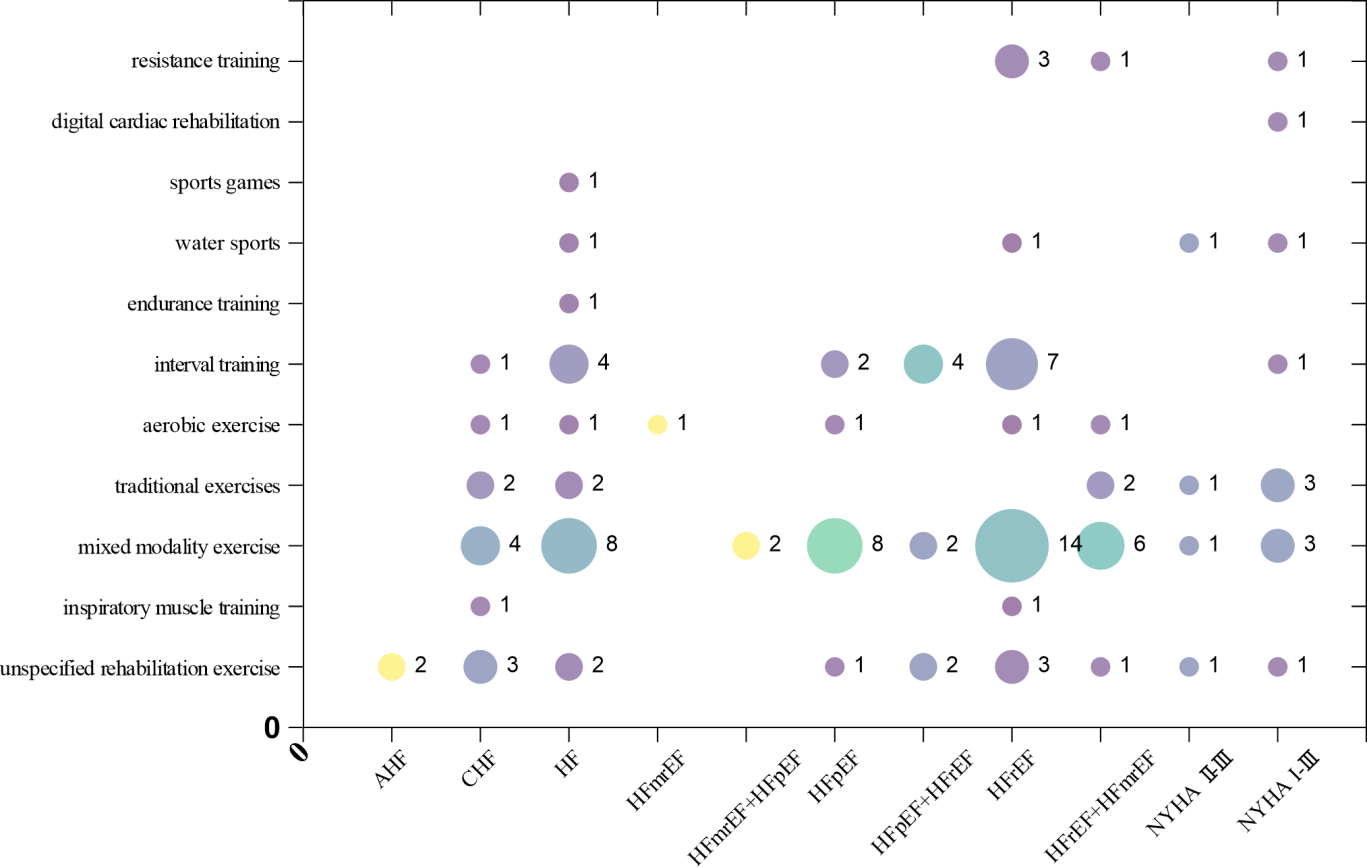
Evidence Map of interventions for exercise interventions in heart failure.

Among the types of heart failure in the studys, HFrEF is the most prevalent (30 articles), followed by the unclassified heart failure population (20 articles). Both the HFmrEF (1 article) and acute heart failure (2 articles) populations receive relatively less attention. Among the studies, the highest number of articles focused on mixed modality exercise(48 articles), followed by those on interval training (19 articles). Among all the type of interventions, the least attention is given to digital cardiac rehabilitation (1 article) and sports games (1 article). The *X*-axis in the bubble chart represents populations with various types of heart failure, while the *Y*-axis represents different type of interventions. The size of the bubbles reflects the number of studies. This distribution is shown in Figure 5.

## 4 Discussion

### 4.1 Main findings

We used an emerging approach called evidence map to visually display information in a user-friendly format. It allows for the systematic analysis of research evidence, progress, and gaps in a specific field, providing a comprehensive overview of the current research situation (36). In conclusion, our findings are as follows: from the final conclusions of the studies, over 80% concluded that the intervention group performed better than the control group, suggesting that exercise is beneficial for heart failure patients. Mixed modality exercise receives the greatest research attention. Existing studies primarily emphasize improving exercise capacity, cardiorespiratory function and quality of life in heart failure patients. The majority of researchers prioritize conducting exercise intervention studies in HFrEF patients.

#### 4.1.1 Mechanisms

The cardioprotective effects of exercise are mediated by multiple mechanisms, including the inhibition of myocardial cell hypertrophy, reduction of inflammation, slowing of myocardial fibrosis progression, decreased likelihood of microvascular dysfunction, and improvement in mitochondrial metabolism and endothelial function. The biological effects of exercise suggest that it may be a promising adjunctive therapy for heart failure (37–40). The greatest benefits with exercise training can be attributed to improvements in cardiorespiratory fitness (41). Long-term exercise training leads to changes in cardiac morphology, including increased end-diastolic volume, ventricular mass, and left ventricular chamber compliance, which contribute to a greater stroke volume and cardiac output during maximal exercise (42). Additionally, exercise training enhances circulating blood volume by approximately 20%–25%. It also promotes nitric oxide (NO) production and bioavailability, improving peak vasodilation of conduit arteries as a functional adaptation, while prolonged training induces shear stress-mediated arterial remodeling as a structural adaptation (43). Exercise training induces various skeletal muscle adaptations that enhance oxygen uptake and utilization. These adaptations include increased capillary density, a higher capillary-to-fiber ratio, a greater proportion of type I (oxidative) muscle fibers (44), and enhanced mitochondrial content and function, driven by an increase in oxidative enzyme capacity (45). Vascular endothelial dysfunction plays a key role in the pathogenesis of atherosclerosis and exercise training improves vascular endothelial function. Exercise training opposes endothelial dysfunction via enhanced endothelial NO synthase and increased NO production and bioactivity, which improves NO-dependent vasodilation of large conduit vessels (46). Exercise helps protect against heart failure through distinct pathways, including the improvement of traditional cardiovascular risk factors such as glycemic control, blood pressure regulation, obesity reduction, and lipid profile optimization (47).

#### 4.1.2 Types of exercise

This study summarized 11 types of exercise suitable for heart failure patients based on existing evidence, with mixed modality exercise being the most commonly studied among them. The combination of aerobic exercise and strength training, known as mixed modality exercise, is particularly suitable for heart failure patients for the following reasons. Mixed modality exercise can be adjusted according to the physical condition and lifestyle of heart failure patients (48). It can be performed at home, in the gym, or outdoors, allowing for flexible choices based on time, location, and physical condition (33). This enhances the feasibility and sustainability of the exercise. For heart failure patients, mixed exercise helps to gradually increase the intensity of exercise, starting with low-intensity aerobic activities and progressing to moderate strength training (49). This approach avoids excessive overload at once and helps improve exercise tolerance. Mixed modality exercise has significant intervention effects, improving cardiovascular function, enhancing muscle strength, and boosting endurance (50). This enables heart failure patients to better cope with daily activities such as walking, climbing stairs, and lifting objects. Interval training is also a widely preferred exercise intervention among heart failure patients (51). Since interval training has a shorter duration and does not require prolonged periods of high-intensity exercise, many patients are more likely to accept and consistently engage in this type of exercise, which relatively improves treatment adherence and long-term outcomes (52).

#### 4.1.3 Outcome indicators

The outcome indicators focused mainly on improving exercise capacity, cardiopulmonary function, and quality of life in heart failure patients. This may be because these indicators are direct outcome measures of exercise intervention, and they are clear to measure, and widely recognized. Given that minimal thresholds of exercise capacity are necessary for one to maintain functional independence throughout the lifespan, exercise capacity is the most efective indicator for improving this parameter (53). Cardiorespiratory fitness is an important factor, as it is linked to the prognosis of heart failure. Maintaining or improving quality of life is an important goal of heart failure therapy, and many patients value better quality of life over greater longevity (54).

A meta-analysis showed that heart failure patients who underwent exercise training had an average increase of 32 meters in their 6-minute walk distance (55). Research indicates that exercise training significantly improves the quality of life scores in heart failure patients (such as the Minnesota Living with Heart Failure Questionnaire), with an average improvement score of −5.5 (56). Regarding hospitalization rates, a systematic review found that heart failure patients who participated in exercise training had a 28% reduced risk of rehospitalization due to heart failure (57). A study found that after a 12-week aerobic exercise intervention, the maximum oxygen consumption (VO2 max) of heart failure patients significantly increased, with an improvement range of 1.7–2.3 ml/kg/min (58).

#### 4.1.4 Heart failure classification

The pathophysiological mechanisms of HFrEF are well understood, and there is a solid research foundation on exercise interventions, making it easier to study the mechanisms of exercise (59). In contrast, the pathophysiology of HFpEF is more complex, and there is less research data on exercise (60). HFrEF patients typically exhibit a significant decline in exercise tolerance (e.g., reduced 6-minute walk test distance and decreased VO₂max). These patients have a lower baseline exercise capacity, so the benefits of exercise training are more pronounced. The safety of exercise in HFrEF patients has been extensively studied, and guidelines (ESC, ACC) strongly recommend exercise training for HFrEF patients (61, 62). The number of studies on stable heart failure is greater than that on acute heart failure.

### 4.2 Limitations and further direction

This study has certain limitations. Firstly, this study is a secondary analysis of previously published literature; therefore, it does not generate new data or viewpoints. Secondly, this study primarily focuses on exercise modalities rather than exercise intensity and duration. Due to significant variations in intensity and duration across different studies, these aspects are not discussed in this research. Thirdly, the quality of the included literature was predominantly low or very low, which may reduce the reliability of the findings. Additionally, this study only included systematic reviews and meta-analyses, excluding original studies, cohort studies, and case-control studies. Consequently, the conclusions of this study may be subject to certain biases. The initial aim of this study was to summarize the optimal exercise prescription for different types of heart failure based on published evidence. Unfortunately, the final results could only present the overall effectiveness of exercise interventions in heart failure patients, summarizing different exercise modalities, outcome measures improved by exercise, and classifications of heart failure patients. Therefore, more randomized controlled trials are needed in the future to fill the existing gaps in evidence and to develop multi-stage optimized exercise strategies for patients at different stages of heart failure, ultimately maximizing improvements in patient care outcomes.

The evidence map results suggest that future research on exercise interventions for heart failure could be improved in the following areas: (1) Standardize Study Design: There is a need for higher-quality primary studies. High-quality studies produce results with higher internal stability and external generalizability, which is essential for replication and dissemination. The evidence map results show that most studies have low or very low quality, and only a few studies are of high quality. (2) Refine Heart Failure Classification: The classification of heart failure types needs to be more precise. Over 17% of the studies in this review did not classify heart failure, leading to imprecise descriptions of the intervention populations. Future research should refine heart failure classifications and increase the study proportion of acute heart failure and patients with NYHA functional class IV heart failure. This would help in comparing the effects of different exercise measures on various types of heart failure. (3) Expand Outcome Indicators: Researchers should focus on a broader range of outcome indicators. The evidence map results show that most studies focused on whether exercise improves cardiopulmonary function, exercise capacity, and quality of life. There was less focus on inflammatory markers, even though studies (63–65) have shown that inflammatory factors are correlated with the severity of heart failure and serve as auxiliary indicators for early diagnosis. Additionally, adverse events related to exercise interventions are often overlooked, and only a few studies (66, 67) reported on exercise-related side effects. It is crucial to provide sufficient evidence regarding the safety of exercise interventions for long-term application and widespread promotion.

## 5 Conclusion

This study systematically reviewed, described, and evaluated evidence related to exercise interventions for patients with heart failure, presenting an overview of the existing evidence in a visually intuitive manner using bubble charts. It systematically demonstrates the quality of studies, research conclusions, and the informational differences among various exercise interventions and outcome indicators, while identifying potential evidence gaps. As seen from the evidence mapping, mixed-mode exercise and interval training are widely applied in heart failure patients, relatively safe, and effective in improving exercise capacity, cardiorespiratory function, and quality of life. In the health education of heart failure patients, these two types of exercise should be prioritized for recommendation. In clinical practice, intervention strategies with strong evidence support should be prioritized, taking into account the patient's baseline condition. This provides valuable reference points and insights for the development of future related research.Exercise interventions have significant advantages for heart failure patients, but there is room for improvement in study quality, heart failure classification, and outcome indicators. Future research should focus on designing more high-quality studies to provide more high-level evidence for this field.

## Data Availability

The original contributions presented in the study are included in the article/Supplementary Material, further inquiries can be directed to the corresponding author.
