# Supplementary material for: Exercise interventions for patients with heart failure: an evidence map
Source: Front Sports Act Living. 2025 Mar 28;7:1557887. doi: 10.3389/fspor.2025.1557887 (PMC11985756; doi:10.3389/fspor.2025.1557887)
Supplement: Supplementary file 1 [file Table1.doc]

**Supplement 1**: **Table 1:**Basic Characteristics of Included Studies

| **Basic Characteristics** | **Number of Articles** | **Percentage** |
| --- | --- | --- |
| **Publication Year** |  |  |
| Before 2010 | 9 | 7.96% |
| 2011-2020 | 59 | 52.21% |
| 2021-2024 | 45 | 39.82% |
| **First Author's Country** |  |  |
| China | 45 | 39.82% |
| Australia | 16 | 14.16% |
| United Kingdom | 13 | 11.50% |
| Brazil | 12 | 10.62% |
| United States | 8 | 7.08% |
| Other Countries (10 countries) | 19 | 16.81% |
| **The Type of Heart Failure** |  |  |
| Acute heart failure (AHF) | 2 | 1.77% |
| Chronic heart failure (CHF) | 12 | 10.62% |
| Heart failure with preserved ejection fraction (HFpEF) | 12 | 10.62% |
| Heart failure with mid-range ejection fraction (HFmrEF) | 1 | 0.88% |
| Heart failure with reduced ejection fraction (HFrEF) | 30 | 26.55% |
| HFmrEF + HFpEF | 2 | 1.77% |
| HFpEF + HFrEF | 8 | 7.08% |
| HFrEF + HFmrEF | 11 | 9.73% |
| NYHA Class I-III | 11 | 9.73% |
| NYHA Class II-III | 4 | 3.54% |
| Unclassified heart failure | 20 | 17.70% |
| **The Type of Intervention** |  |  |
| Aerobic Exercise | 6 | 5.31% |
| Interval Training | 19 | 16.81% |
| Resistance Training | 5 | 4.42% |
| Endurance Training | 1 | 0.88% |
| Traditional Exercises | 10 | 8.85% |
| Aquatic Exercise | 4 | 3.54% |
| Exercise Games | 1 | 0.88% |
| Inspiratory Muscle Training | 2 | 1.77% |
| Mixed Exercise | 48 | 42.48% |
| Digital Remote Rehabilitation | 1 | 0.88% |
| Rehabilitation without specified exercise type | 16 | 14.16% |
| **Outcome Indicators** |  |  |
| Cardiopulmonary Function | 61 | 53.98% |
| Exercise Capacity | 89 | 78.76% |
| Quality of Life | 48 | 42.48% |
| Inflammatory Markers | 5 | 4.42% |
| TCM Symptom Scores | 1 | 0.88% |
| Psychological Status | 4 | 3.54% |
| Other (mortality, hospitalization rate, morbidity) | 12 | 10.62% |
| **Bias Risk Assessment Tools** |  |  |
| Cochrane Bias Risk Tool | 60 | 53.10% |
| Jadad Scale | 10 | 8.85% |
| JBI Score | 3 | 2.65% |
| PEDro Scale | 25 | 22.12% |
| TESTEX Scale | 13 | 11.50% |
| Downs and Black Quality Index | 1 | 0.88% |
| Delphi List Score | 1 | 0.88% |
| **Intervention Details** |  |  |
| Described Intervention Time, Intensity, Frequency | 104 | 92.04% |
| Not Described | 9 | 7.96% |
| **Efectiveness Evaluation** |  |  |
| Intervention Group Superior | 95 | 84.07% |
| No Significant Difference | 18 | 15.93% |

**Supplement 2: Search strategy**

**1.PubMed database**

#1 "heart failure" [MeSH]

#2 "heart failure" OR "cardiac failure" OR " heart decompensation" OR " myocardial failure " OR " congestive heart failure " OR "CHF" OR "heart decompensation" OR "le­-sided heart failure" OR "HFrEF" OR "HFpEF" OR "HFmrEF" [Title/Abstract]

#3 #1 OR #2

#4 " exercise " [MeSH]

#5 " exercis* " OR " activity scheduling " OR " fitness " OR " cycling " OR " athletic support " OR " aerobic training " OR " running " OR " jogging " OR " walking " OR " swim*" OR " physical exercise " OR " physical performance" OR " physical well being" OR " high-intensity interval training" OR " plyometric exercise" OR " resistance training" OR "endurance training" [Title/Abstract]

#6 #4 OR #5

#7 "Meta-analysis" OR "systematic review"[Title/Abstract]

#8 #3 AND #6 AND #7

**2. CNKI Chinese database**1.心力衰竭

2.心衰

3.心脏衰竭

4.心功能不全

5.HF

6.运动

7.锻炼

8.系统评价

9.Meta分析

10.系统综述

11.荟萃分析

12.元分析

13.系统分析

14. OR/ 1-5 15. OR/6-7

16. OR/8-13 17. 14 AND 15 AND 16

Similar searches were performed in the remaining databases.
